# Supplementary material for: The causal relationship between the human gut microbiota and pyogenic arthritis: a Mendelian randomization study
Source: Front Cell Infect Microbiol. 2024 Nov 26;14:1452480. doi: 10.3389/fcimb.2024.1452480 (PMC11629706; doi:10.3389/fcimb.2024.1452480)
Supplement: Supplementary file 1 [file DataSheet1.zip › mendelian test/ebi-a-GCST90027597.csv_forest.pdf]

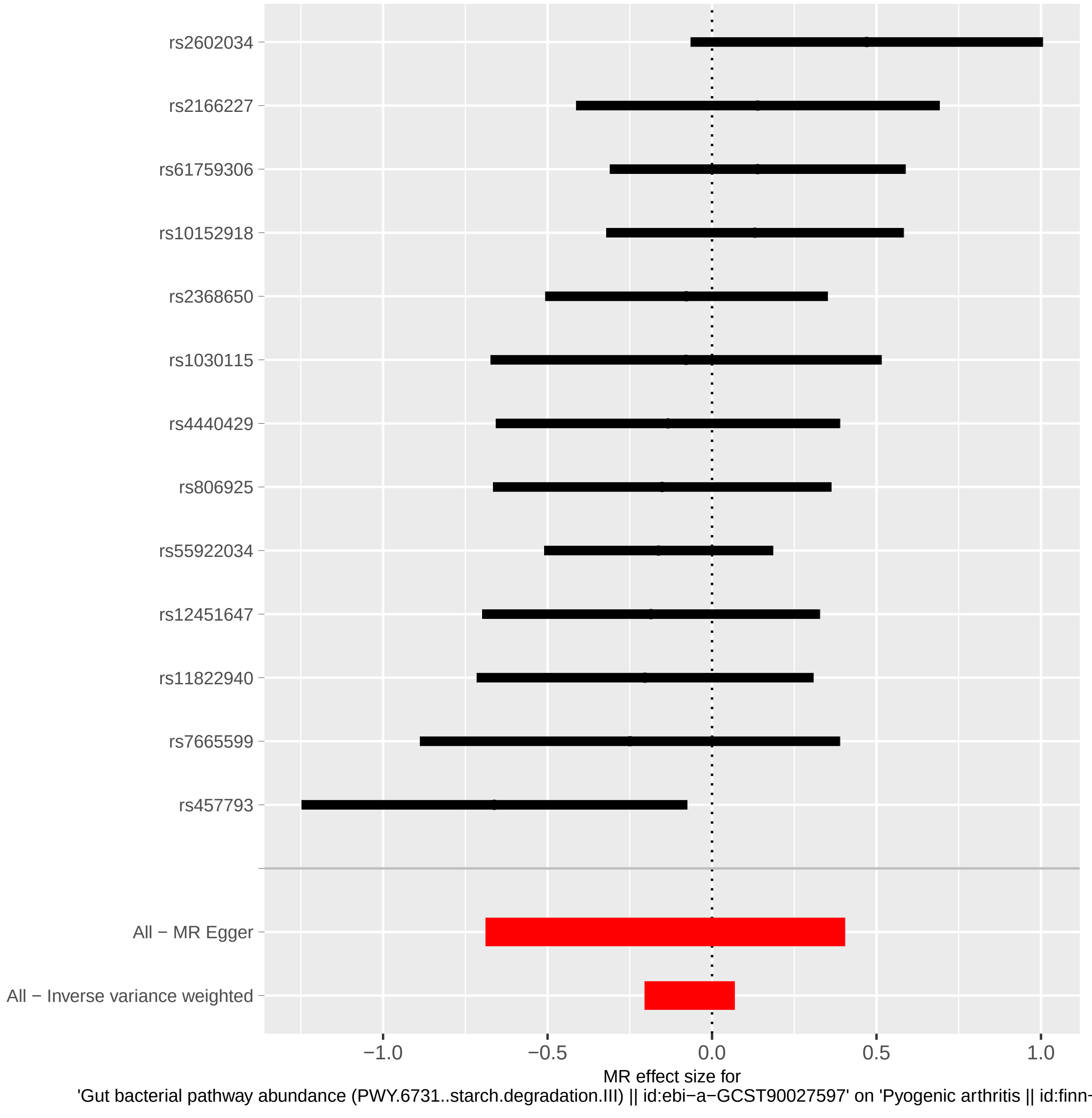
rs2602034
rs2166227
rs61759306
rs10152918
rs2368650
rs1030115
rs4440429
rs806925
rs55922034
rs12451647
rs11822940
rs7665599
rs457793
All − MR Egger
All − Inverse variance weighted
−1.0
−0.5
0.0
0.5
1.0
MR effect size for
'Gut bacterial pathway abundance (PWY.6731..starch.degradation.III) || id:ebi−a−GCST90027597' on 'Pyogenic arthritis || id:finn−
